# 核苷酸还原酶M1在晚期非小细胞肺癌治疗和预后中的作用

**DOI:** 10.3779/j.issn.1009-3419.2015.06.09

**Published:** 2015-06-20

**Authors:** 家伟 田, 淑华 韩

**Affiliations:** 1 210009 南京，东南大学医学院 Medical College, Southeast University, Nanjing 210009, China; 2 210009 南京，东南大学附属中大医院呼吸科 Department of Respiration, Zhongda Hospital Affiliated to Southeast University, Nanjing 210009, China

**Keywords:** 肺肿瘤, 核糖核苷酸还原酶类, 个体化治疗, 预后, Lung neoplasms, Ribonucleotide reductases, Individualized treatment, Prognosis

## Abstract

肺癌是最常见和死亡率最高的恶性肿瘤之一，75%-80%为非小细胞肺癌（non-small cell lung cancer, NSCLC）。且绝大部分患者就诊时已处晚期，失去了根治性手术或放疗的机会，化疗是主要的治疗手段。但由于肿瘤生物学行为的多样性和对化疗药物的耐药等，使得晚期NSCLC的化疗不容乐观。近年来，分子标志物的个体化化疗在晚期NSCLC的应用，使得这些患者生存期有所延长，生活质量有所提高。针对晚期NSCLC，根据分子标志物选择合适的药物，达到个体化化疗是临床中所要面对和解决的问题。本文就核苷酸还原酶M1（ribonucleotide reductase subunit 1, RRM1）在晚期NSCLC治疗和预后中的作用做一简要综述。由RRM1指导的个体化化疗并不能作为晚期NSCLC的常规决策，还需进一步研究。

## Abstract (en)

Lung cancer is one of the most common and highest mortality rates malignant tumors, further, 75%-80% is non-small cell lung cancer (NSCLC). For the majority of patients, lost the chance of radical operation or radiotherapy, chemotherapy is the main treatment. However, because of the diversities of tumor behavior and drug-resistant, the chemotherapy of advanced NSCLC is not optimistic. In recent years, with the application of molecular markers for individual chemotherapy, these patients have achieved prolong life and improved life quality. Individualized chemotherapy based on molecular markers to select the appropriate drug is the problem that needs to be solved. The paper gives a brief review on the role of ribonucleotide reductase subunit 1 (RRM1) in the treatment and prognosis of advanced NSCLC. Individualized chemotherapy by RRM1 can't become commonplace for advanced NSCLC and needs further research.

肺癌为临床常见恶性肿瘤，据世界卫生组织国际癌症研究机构最新的2012年统计资料显示，当年新发肺癌180万，占所有新发恶性肿瘤的12.9%，死亡159万，占所有因恶性肿瘤死亡的19.4%^[1]^。可见，其发病率和病死率之高，而且还在不断上升^[2]^，更让人担忧的是其5年生存率，在男性仅为6%-14%，女性仅为7%-18%^[3]^。肺癌患者中75%-80%为非小细胞肺癌（non-small cell lung cancer, NSCLC），就诊时多已处晚期，失去了根治性手术或放疗的机会，化疗是主要的治疗手段。但由于肿瘤生物学行为的多样性和对化疗药物的耐药等因素，使得晚期NSCLC的化疗结果不容乐观。近年来，分子标志物的个体化化疗在晚期NSCLC的应用，使得这些患者生存期有所延长，生活质量有所提高，如RRM1与吉西他滨（Gemcitabine, Gem）耐药及治疗预后相关，核苷酸切除修复交叉互补组（excision repair cross-complementing 1, ERCC1）和人类乳腺癌易感基因（breast cancer susceptibility gene breast cancer 1, BRCA1）与铂类（platinum, P）相关，胸苷酸合成酶（thymidylate synthase, TS）与培美曲塞（pemetrexed），Ⅲ型β-微管蛋白（class Ⅲ β-tubulin, TUBB3）与紫杉醇（Taxol, Tax）相关，表皮生长因子受体（epidermal growth factor receptor, EGFR）突变与EGFR-酪氨酸激酶抑制剂（tyrosine kinase inhibitor, TKI），ALK基因重排与克唑替尼等^[4]^。因此，针对晚期NSCLC，根据分子标志物选择合适的药物，达到个体化化疗是临床中所要面对和解决的问题^[5]^。本文就RRM1在晚期NSCLC治疗和预后中的作用做一简要综述。

## 1 RRM1概况

RRM1是核苷酸还原酶（ribonucleotide reductase, RR）的M1亚基。DNA的合成和损伤修复都需要RRM1的参与。RRM1与晚期NSCLC Gem化疗和预后存在相关性。高表达RRM1（RRM1 positive, RRM1+）使得肿瘤细胞具有很强的合成和损伤修复能力，可造成对Gem的耐药和影响预后。临床中常用免疫组化（immunohistochemical, IHC）和PCR检测RRM1，由于考虑到转录后加工的影响和现有IHC所用抗体不能特异地检测出相关亚型，两者优劣多无定论^[6-8]^。同时，肿瘤组织和外周血检测无明显差异^[9]^，这对无法获取肿瘤组织标本的晚期NSCLC个体化化疗意义重大。

RR由大的调节亚基RRM1和小的催化亚基RRM2/RRM2B组成。RRM2B是p53诱导的一个亚基，可取代RRM2发挥作用。目前的研究主要集中于RRM1，除了RRM2仅起到催化作用外，还因为RRM2仅在DNA合成的G_1_期-S期才可被检测到，半衰期3 h，而RRM1出现在DNA合成的全部时相，半衰期可达15 h^[10]^。但也有研究^[11]^表明RRM2也是抗癌药物的一个重要靶点。RRM1编码基因位于染色体的11p15.5，在许多肿瘤细胞中呈现为杂合丢失^[12]^。当两个等位基因都存在时会起到抑制肿瘤的作用；某一个等位基因缺失时就不再具有抑癌作用，且细胞转为癌细胞；两个等位基因的全缺失时RR不能激活，细胞死亡；纯合子少见^[13]^。RRM1是核苷酸的结合位点，能特异识别结合底物和发生变构调节RR。RR在DNA的合成中负责催化二磷酸核苷酸（ribonucleoside diphosphate, NDP）向为二磷酸脱氧核苷酸（deoxyribonucleoside diphosphate, dNDP）的转化^[13]^，后者是DNA合成和损伤修复的原料。因此，RRM1既具有抑制肿瘤的作用，同时还有促进DNA合成和损伤修复的功能（图 1）。

**1:**
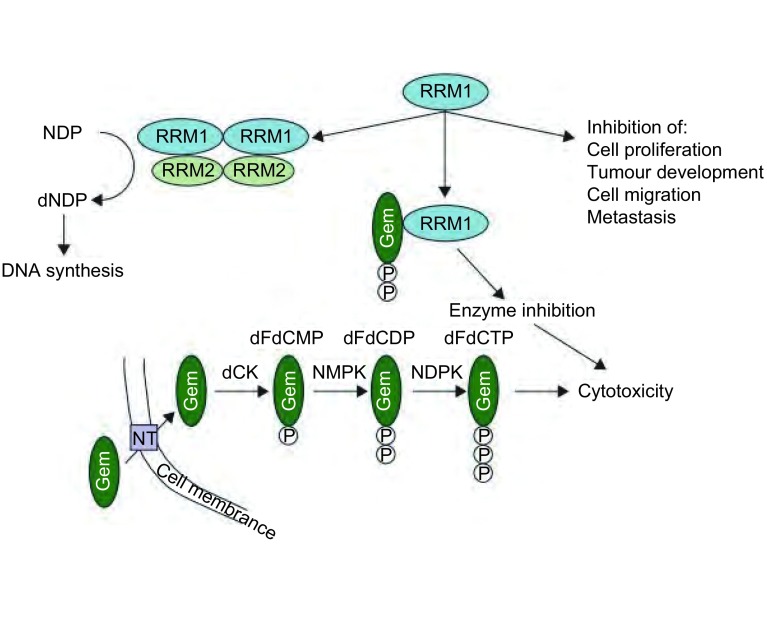
RRM1在含Gem化疗方案中的作用机制。RRM1作为RR的M1亚基，使NDP转化为dNDP，参与DNA的合成。杂合不缺失等位基因的RRM1可起到抑制肿瘤的作用。Gem作为核苷类似物，在细胞内代谢为具有活性的双氟二磷酸脱氧胞苷（Gem diphosphate, dFdCDP）和双氟三磷酸脱氧胞苷（Gem triphosphate, dFdCTP）。dFdCDP结合于RRM1核苷酸的结合位点，抑制了RR的活性；dFdCTP掺合到DNA中使其合成受阻。两者共同发挥Gem的细胞毒作用。注：本图已获得版权所有者Elsevier使用许可[Jordheim LP, Seve P, Tredan O, *et al*. The ribonucleotide reductase large subunit (RRM1) as a predictive factor in patients with cancer. Lancet Oncol, 2011, 12(7): 693-702.]。 The mechanism of RRM1 in containing Gem chemotherapy. RRM1 is the M1 subunit of RR, converting NDP into dNDP, participates in DNA synthesis. RRM1, heterozygous and not loss its alleles, has the function of inhibiting the growth of tumours. Gem as a nucleoside analogues is metabolized to active dFdCDP and dFdCTP. dFdCDP binds to its sites on the RRM1, reducing RR activity; dFdCTP gets involved in DNA synthesis and makes the process blocked. Both of them work together as the cytotoxicity of Gem. RRM1: ribonucleotide reductase subunit 1; RR: ribonucleotide reductase; NDP: ribonucleoside diphosphate; dNDP: deoxyribonucleoside diphosphate; Gem: Gemcitabine; dFdCDP: Gem diphosphate; dFdCTP: Gem triphosphate. Note: With permission from the copyright holder Elsevier [Jordheim LP, Seve P, Tredan O, *et al*. The ribonucleotide reductase large subunit (RRM1) as a predictive factor in patients with cancer. Lancet Oncol, 2011, 12(7): 693-702].

## 2 RRM1疗效预测及预后

### 2.1 RRM1在含Gem化疗方案中的作用机制

Gem是核苷类似物，属抗代谢类抗癌药。Gem由膜上的核苷转运体（nucleoside transporter, NT）介导进入细胞。在脱氧胞苷激酶（deoxycytidine kinase, dCK）、核苷一磷酸激酶（nucleoside monophosphate kinase, NMPK）和核苷二磷酸激酶（nucleoside diphosphate kinase, NDPK）的作用下相继转化为双氟一磷酸脱氧胞苷（Gem monophosphate, dFdCMP）和具有活性的dFdCDP、dFdCTP。dFdCDP结合于RRM1核苷酸的结合位点，抑制了RR的活性，dFdCTP掺合到DNA中使其合成受阻，两者共同发挥Gem的细胞毒作用（图 1）。对于Gem的获得性耐药，则是细胞在受到Gem损伤后使RR过表达以利于修复，正常的三磷酸脱氧胞苷（dCTP）在细胞中含量相应增加，dFdCTP相对地很少再替代dCTP掺合到DNA中使其合成受阻。另一耐药机制则是通过使可介导Gem在胞内发挥作用的NT和dCK等减少，同时使如胞嘧啶核苷脱氨酶和5’-核苷酸酶等可发挥降解dFdCMP、dFdCDP和dFdCTP作用的酶增加所致^[14, 15]^。

### 2.2 RRM1在晚期NSCLC的治疗与预后

晚期NSCLC可选择手术切除，但对于绝大多数来说，在诊断时已属晚期，丧失了手术机会。晚期NSCLC除可依据相应的分子标志物选择靶向治疗外，化疗仍是其基础治疗^[5]^。以铂类为基础联合第三代细胞毒类药，如Gem、Tax、多西他赛（Docetaxel, Doc）和长春瑞滨（vinorelbine）等，作为一线选择方案可延长晚期NSCLC的生存期并提高生存质量^[16]^。但是，临床中对于联合化疗具体药物的选择，常常是医生根据自己的临床经验和个人偏好，这就造成了疗效的很大变异，而以分子标志物表达的不同选择的个体化治疗可减少化疗药物耐药和改善预后^[17, 18]^。Zhang等^[19]^就通过其前瞻性研究证实了依据RRM1、ERCC1和TUBB3选择的个体化化疗的晚期NSCLC患者相比于统一采用铂类联合Gem的化疗有很好的化疗反应、中位无进展生存期（progression free survival, PFS）和1年生存率。其对85例Ⅲb期或Ⅳ期的晚期NSCLC患者，41例已知晓分子标志物情况的分为A组，44例不知晓的分为B组。A组依据ERCC1低表达（ERCC1 negative, ERCC1-）接受以铂为基础的联合化疗（Doc/Tax/Gem/vinorelbine/pemetrexed+P）；RRM1-的接受Gem单药或以Gem为基础的联合；TUBB3高表达（TUBB3 positive, TUBB3+）的不接受Tax；由于pemetrexed不推荐用于鳞癌，RRM1+和TUBB3+排除在外等原则，被分为ERCC-RRM-（Gem+P）、ERCC-RRM1+TUBB3-（Tax/Doc+P）、ERCC-RRM1+TUBB3+（pemetrexed+P）、ERCC+RRM1-TUBB3-（Gem+Tax）、ERCC+RRM1-TUBB3+（Gem）、ERCC+RRM1+TUBB3-（Tax+Doc）和ERCC+RRM1+TUBB3+（pemetrexed）7个亚组。B组统一选择Gem+P的联合化疗。随访发现A组完全缓解（complete response, CR）和部分缓解（partial response, PR）占56.1%，明显高于B组的31.8%（*P*=0.024），且在PFS（5.2 mo *vs* 4.1 mo, *P*=0.026）和1年生存率（65.9% *vs* 40.9 %, *P*=0.021）上也明显存在优势。RRM1与以铂类联合Gem的化疗相关，大量的基础和临床研究^[20-22]^均表明RRM1-的对Gem敏感，耐药发生少，有较好的预后。RNA干扰（RNAi）是肿瘤研究的重要技术手段，利用RNAi使*RRM1*基因沉默后，那些对Gem耐药并RRM1+的肿瘤细胞RRM1蛋白产生也相应减少。Wonganan等^[23]^就给予小鼠这种RRM1特异的干扰RNA，观察到小鼠体内RRM1+的肺癌细胞生长受到了抑制，而且转而对Gem变得敏感。Li等^[21]^对40例入组的晚期NSCLC中34例满足条件的患者进行分析，RRM1-的较RRM1+对以铂类联合Gem的化疗反应（52.9% *vs* 5.9%, *P*=0.007）和预后（15.5 mo *vs* 12.0 mo, *P*=0.046）好。Dong等^[24]^用IHC检测RRM1回顾性分析了680例晚期NSCLC，229例接受了以铂类为基础的联合化疗，在使用铂类联合Gem的患者中，RRM1-在疾病控制率（disease control rate, DCR）和PFS上都存在显著差异（DCR 78.8% *vs* 55.2%, *P*=0.041; PFS 8.8 mo *vs* 7.6 mo, *P*=0.01），同时在性别、吸烟史和TNM分期的多因素分析中，RRM1是影响PFS的独立影响因素（95%CI: 1.135-2.907, *P*=0.013）。国外也有学者研究发现，在以含Gem化疗的晚期NSCLC中，RRM1-患者在总生存期[（overall survival, OS）, 12.9 mo *vs* 5.1 mo, *P*=0.022]和DCR[PR+疾病稳定（stable disease, SD）, 56% *vs* 23%, *P*=0.053]均优于RRM1+^[25]^。但也有少部分研究表明不存在上述相关，一项针对RRM1的Ⅲ期临床试验^[26]^就曾报道，选择Gem的RRM1-在DCR、PFS和OS没有获益。另一项根据RRM1和ERCC1表达情况个体化化疗的国际Ⅲ期随机临床试验^[27]^也得出，个体化治疗组较对照在PFS（6.1 mo *vs* 6.9 mo, *Log-rank*, *P*=0.181）和OS（11.0 mo *vs* 11.3 mo, *Log-rank*, *P*=0.66）上无差异。之所以会出现上述不一致的研究结论，有学者^[28, 29]^认为可能与*RRM1*的基因多态性相关。近来的的一项*meta*分析表明，RRM1-的晚期NSCLC对于以铂类联合Gem的化疗有很好的化疗反应（OR=0.31, 95%CI: 0.21-0.45, *P* < 0.000, 01），有更长的PFS（2.64 mo, 95%CI: 0.39-4.89, *P*=0.02）和存活期（3.94 mo, 95%CI: 2.15-5.73, *P* < 0.000, 1）^[30]^（表 1）。

**1:** 本文中所涉及的一些关于RRM1在晚期NSCLC中的研究 Some studies about the role of RRM1 in the advanced NSCLC

	Other markers studied	*n*	Treatment	Findings
Protein level^[19]^	ERCC1, TUBB3	85	A group (44): ERCC-RRM- (Gem+P), ERCC-RRM1+TUBB3- (Tax/Doc+P), ERCC-RRM1+TUBB3+ (pemetrexed+P), ERCC+RRM1-TUBB3- (Gem+Tax), ERCC+RRM1-TUBB3+ (Gem), ERCC+RRM1+TUBB3- (Tax+Doc), ERCC+RRM1+TUBB3+ (pemetrexed). B group(41): Gem+P	The overall response rate, defined as CR plus PR, was 56.1% for group A, significantly higher than that in group B (31.8%; *P*=0.024). The PFS and the 1-yr survival rate was 5.2 mo and 65.9% for group A, significantly longer and higher than that of group B (4.1 mo, *P*=0.026; 40.9%, *P*=0.021).
mRNA expression^[20]^	BRCA1	94	RRM-BRCA- (Gem+P), RRM1-BRCA1+ (Gem+vinorelbine), RRM1+BRCA1- (Doc+P), RRM1+BRCA1+ (vinorelbine+P).	The response rates in the Gem+P, Doc+P and vinorelbine+P groups were 42.9%, 36.7% and 27.9%, respectively (*P*=0.568). The PFS was 5.6, 5.0 mo, 4.8 mo (*P*=0.975), respectively, and the OS was 12.5 mo, 11.0 mo, 9.7 mo (*P*=0.808), respectively.
mRNA expression^[21]^	ERCC1, BRCA1	34	Gem+P	The response rate in the RRM1- group was significantly greater than in the RRM1+ group (52.9% *vs* 5.9%, *P*=0.007).
Protein level^[22]^	ERCC1, TUBB3	86	The study group received chemotherapy (P, Gem, Tax and pemetrexed) under the guidance of molecular markers (ERCC1, RRM1 and TS). The control group received vinorelbine.	The PFS of the study group and the control group was 4.0 mo (95%CI: 3.1-4.9) and 3.0 mo (95%CI: 2.4-3.6) respectively. The difference being statistically significant (*χ*^2^=4.750, *P*=0.029). The differences of the objective response rate and DCR being not significant.
Protein level^[24]^	-	299	Platinum doublet chemotherapy	In patients receiving gemcitabine-based therapy, the DCR and PFS of RRM1- was significantly higher than RRM1+ (*P*=0.041 and *P*=0.01, respectively)
Protein level^[25]^	-	40	Platinum doublet chemotherapy	The OS of RRM1+ was significantly shorter than RRM- (5.1 mo *vs* 12.9 mo, *P*=0.022). DCR (PR+SD) of the RRM1+ was significantly lower than that of RRM1- (23% *vs* 56%, *P*=0.053).
NSCLC: non-small cell lung cancer; CR: complete response; DCR: disease control rate; PFS: progression-free survival; PR: partial response; SD: stable disease; P: platinum; Tax: taxol; Gem: gemcitabine; Doc: docetaxel.				

## 3 小结

RRM1作为RR的M1亚基，负责NDP向dNDP的转化，从而参与DNA的合成和损伤修复。Gem为核苷类似物，可结合于RRM1的核苷酸结合位点，抑制RR，阻碍DNA的合成。目前已有大量的研究表明，RRM1-的晚期NSCLC对以Gem联合的化疗有较好的化疗反应和预后，但也有少部分研究与其正好相反，这可能与*RRM1*的基因多态性相关。由RRM1指导的个体化化疗并不能作为晚期NSCLC的决策常规，还需进一步研究。化疗是晚期NSCLC的基础治疗，不同个体对化疗的敏感性和预后存在差异，根据分子标志物表达的不同而选择的个体化治疗方案可减少化疗耐药和改善预后，这仍然是晚期NSCLC治疗方面研究的重要内容之一。
